# Estimating and validating disability-adjusted life years at the global level: a methodological framework for cancer

**DOI:** 10.1186/1471-2288-12-125

**Published:** 2012-08-17

**Authors:** Isabelle Soerjomataram, Joannie Lortet-Tieulent, Jacques Ferlay, David Forman, Colin Mathers, D Maxwell Parkin, Freddie Bray

**Affiliations:** 1 Department of Public Health, Erasmus MC, Rotterdam, the Netherlands; 2 Department of Global Health and Population, Harvard School of Public Health, Boston, USA; 3 Section of Cancer Information, International Agency for Research on Cancer, 150 Cours Albert Thomas, Lyon, 69372, France; 4 World Health Organization, Geneva, Switzerland; 5 Clinical Trial Service Unit and Epidemiological Studies Unit, University of Oxford, Oxford, UK

**Keywords:** Years of life live with disability, Years of life lost, Disability-adjusted life years, Cancer, Global estimates

## Abstract

**Background:**

Disability-adjusted life years (DALYs) link data on disease occurrence to health outcomes, and they are a useful aid in establishing country-specific agendas regarding cancer control. The variables required to compute DALYs are however multiple and not readily available in many countries. We propose a methodology that derives global DALYs and validate variables and DALYs based on data from various cancer registries.

**Methods:**

We estimated DALYs for four countries (Norway, Bulgaria, India and Uganda) within each category of the human development index (HDI). The following sources (indicators) were used: Globocan2008 (incidence and mortality), various cancer registries (proportion cured, proportion treated and duration of disease), treatment guidelines (duration of treatment), specific burden of disease studies (sequelae and disability weights), alongside expert opinion. We obtained country-specific population estimates and identified resource levels using the HDI, DALYs are computed as the sum of years of life lost and years lived with disabilities.

**Results:**

Using mortality:incidence ratios to estimate country-specific survival, and by applying the human development index we derived country-specific estimates of the proportion cured and the proportion treated. The fit between the estimates and observed data from the cancer registries was relatively good. The final DALY estimates were similar to those computed using observed values in Norway, and in WHO’s earlier global burden of disease study. Marked cross-country differences in the patterns of DALYs by cancer sites were observed. In Norway and Bulgaria, breast, colorectal, prostate and lung cancer were the main contributors to DALYs, representing 54% and 45%, respectively, of the totals. These cancers contributed only 27% and 18%, respectively, of total DALYs in India and Uganda.

**Conclusions:**

Our approach resulted in a series of variables that can be used to estimate country-specific DALYs, enabling global estimates of DALYs and international comparisons that support priorities in cancer control.

## Background

Cancer is one of the major causes of morbidity and mortality globally, with 12.7 new cancer cases and 7.6 million cancer deaths worldwide in 2008 [1,2]. Despite successes in the prevention, early detection and screening of cancers in some populations, the number of new cancer cases is increasing globally, partly due to population ageing and growth, but also as a result of changing prevalence and distribution of the major risk determinants for cancer in different populations. As a result, cancer is projected to become one of the main causes of death in low, middle and high income countries in the coming decades [1].

In describing cancer patterns and trends as a means to plan and evaluate cancer control policies, incidence, mortality and survival are considered the standard set of indicators. This study provides a methodological framework for estimating an indicator that integrates the above measures with metrics related to non-fatal outcomes. Disability-adjusted life years (DALYs) measure loss of health as a result of illness in the population relative to the ideal scenario where everyone in the population lives into old age in full health [3]. It is a time-based measure that combines the time lost due to premature mortality (years of life lost, or YLLs) and the duration of disability (years of life lived with a disability, or YLDs) in survivors. One lost DALY equates to one lost year of “healthy” life, either as a result of premature death from the disease, or because of disease-related illnesses or disability [3]. Survival for a number of common cancers has been increasing in the last decades, resulting in an ever-increasing number of survivors [4], some living with cancer sequelae. By estimating disability-adjusted life years (DALYs), two key components of the burden of cancer are captured: one related to premature mortality, the other to the loss of ‘healthy’ life years related to the morbidity that follows a diagnosis of cancer [3].

The paper aims to provide an overview of methodological approaches to calculate sex-specific DALYs for 27 cancer types based on the modeling of indicators derived from numerous epidemiological histories for each of the common cancer sites. Cancer-specific DALYs have been previously estimated in a global or national context [1,5,6]. Besides taking a country-specific approach to build up the global picture, we propose a unified framework that takes into account the natural history of each cancer site under study. The key parameters - incidence, mortality, survival and the proportion cured / treated are estimated, based on the most recent population-based data or using results from specific studies. The basic calculation methods developed for the global burden of disease (GBD) study [3] were used and adapted. Finally we validated the general disease model on the basis of an assumed set of natural histories for each of the common cancer sites for four countries, one within each category of the human development index (HDI): Norway, Bulgaria, India and Uganda.

## Methods

To calculate DALYs [3], YLLs and YLDs are independently calculated and then combined in a single summary measure (Formula 1).

(1) DALY⋅=YLL⋅+⋅YLD

YLLs were calculated by multiplying the number of cancer-specific deaths at a given age by the standard life expectancy for that age (or age group, Formula 2). As has been done in earlier global burden of disease project, we used the model life-table West with a life expectancy at birth of 82.5 years for women and 80 years for men [7]. We used the life expectancy at the mid-point for each age group (0–14, 15–39, 40–44, 45–49,… , 70–74, 75 and over), corresponding to the age-specific mortality data available on a global basis (see below).

(2) $$\text{YLL}=\sum_{X}d_{x}{e^{*}}_{x}$$

where *d* = death, *x* = age, e* standard expectation of life at age *x*.

YLDs were derived as the product of the number of new cases, the average duration of the disability and disability weightings for the condition (or disease state, Formula 3). Disability weights represent a value preference that scales a condition or state from 0 (full health) to 1 (death) [3]. To allow comparison between countries, YLLs and YLDs were age standardized by the direct method, using the world standard population [8].

(3) $$\text{YLD}=\sum_{x_{,}y}i_{x_{,}y}dw_{y}d_{x_{,}y}$$

where *i* = incidence, *x* = age, *y* = disease phase, *dw* = Disability weight, *d* = duration of disability.

**Figure 1:**
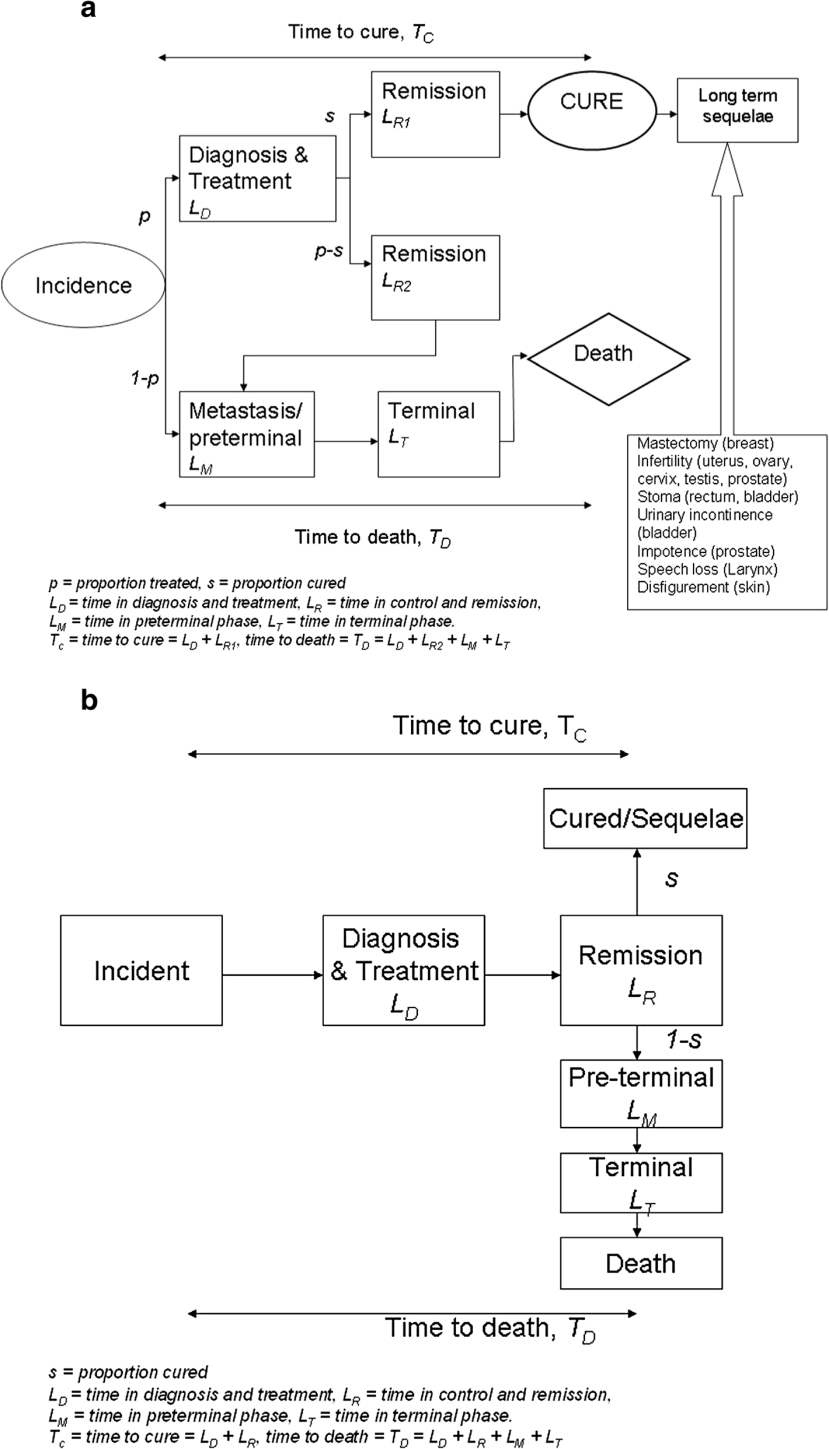
**a: Three-stage natural disease history for cancer.b**: Two-stage disease history model.

The general form of the proposed natural history model for cancer is illustrated in Figure 1. We assumed three possible pathways for newly-diagnosed cancer cases:

I.  Those who were treated (*p*) and then cured (*s*) from cancer, underwent a period of disability during the primary diagnosis and therapy phase (duration = *L*_*D*_), and a period of disability during remission (*L*_*R1*_)_*,*_ during which patients underwent intensive follow-up to detect recurrence or dissemination. A proportion of treated patients who were considered cured continued to live permanently with one (or more) cancer sequelae.

II.  Those who died from cancer after treatment (*ps*), underwent a period of disability in the following phases: (i) primary diagnosis and therapy (*L*_*D*_), (ii) the remission period *L*_*R2*_ (for which the duration was estimated as the time between treatment and death minus 4 months) [5], (iii) the pre-terminal phase *L*_*M*_ and (iv) the terminal phase *L*_*T*_. *L*_*M*_ constitutes the phase where cancer has disseminated and was set to last for 3 months [5]. The terminal phase *L*_*T*_, where patients are at the final stages of living, was uniformly set for 1 month [5].

III.  Those who did not receive treatment (*1-p*) underwent a period of disability during the pre-terminal (*L*_*M*_) and terminal phases (*L*_*T*_). This group was considered to comprise advanced cancer cases, who would have received palliative treatment only.

On the basis of this natural history model and the parameters contained within, we estimated DALYs for 27 cancers. In this paper we present and validate DALYs for four countries, one within each category of the HDI: Norway, Bulgaria, India and Uganda, in 2008. The main sources of data and the estimation methods are set out below.

### Cancer incidence and mortality

The incident number of cancer cases and deaths according to sex and age were extracted from GLOBOCAN 2008 [2] for 27 cancer sites: lip and oral cavity (ICD-10 C00-08), nasopharyx (C11), other pharynx (C09-10, C12-14), oesophagus (C15), stomach (C16), colorectum (C18-21), liver (C22), gallbladder (C23-24), pancreas (C25), larynx (C32), trachea, bronchus and lung (C33-34), melanoma of skin (C43), Kaposi sarcoma (C46), breast (C50), cervix uteri (C53), corpus uteri (C54), ovary (C56), prostate (C61), testis (C62), kidney (C64-66), bladder (C67), brain, nervous system (C70-72), Hodgkin lymphoma (C81), non-Hodgkin lymphoma (C82-85, C96, B21.1-2), multiple myeloma (C88, C90) and leukemia (C91-95)). Age was stratified into 10 groups (0-14, 15-39, 40-44, 45-49, …, 70-74, 75 and over).

### Proportion cured, proportion treated and proportion with sequelae

#### Proportion cured (s)

The proportion of patients statistically cured from cancer is reached when a group of patients have (almost) the same mortality as the general population [9]. To determine the country and cancer-specific cured proportions, we firstly estimated case fatality as the ratio of mortality (the number of deaths, *M*) to incidence (number of new cases, *I*), which is also a proxy for 5-year survival as 1 – *(M/I)*[10,11], based on the GLOBOCAN estimates. Secondly, we obtained a smoothed function of the estimated survival regressed according to the levels of the human development index (HDI) [12] via a log-linear regression model. Here HDI was chosen because it is a composite of various factors including investment in health infrastructure, and is expected to predict survival. Fourthly, we calculated the ratios of the proportion cured to 5-year survival from a published report from the Cancer Registry of Norway [13]. Finally the ratios (proportion cured:survival) were applied to the country-specific fitted values of survival to provide corresponding country-specific estimates of the proportion cured.

#### Proportion treated (p)

This measure was estimated as the percentage of patients who received either surgery, chemotherapy, radiotherapy or a combination of two or more of the aforementioned treatments. We applied the ratio of the proportion treated (from the Cancer Registry of Norway (for solid tumors) and of Ireland (for Kaposi sarcoma and haematological malignancies)) to the reported proportion cured, [13] to the estimated proportion cured derived as described above, to obtain country-specific proportions treated.

#### Proportion of survivors living with sequelae

Sequelae among survivors are commonly related to the cancer-specific treatment. Estimating the distribution of survivors who are living with sequelae requires systematic assessment of the available evidence on treatment types, the survival among specific treatment group, as well as the duration and severity of the disability. These data are generally not available; and where they were, were mainly from high-resource countries with highly-developed cancer registration systems. We therefore could only obtain for a number of limited cancer sites, a set of disabling sequelae (Table 1). The final estimates for the proportion of survivors living with sequelae were derived by combining several information sources, including cancer registries, peer-reviewed literature [14-20] and oncological treatment guidelines [21]. For example: for ovarian, cervical and corpus uteri cancers, the standard treatment is hysterectomy; thus all cured patients were assumed to have had this treatment, with infertility as a sequelae.

**Table 1:** Proportion with sequelaes and disability weights for selected cancer sites and data sources

**Sites/sequelae**	**Source of data**	Proportion*	**Disability weights**	**Remarks**
**Rectum**				
Stoma	Morris E et al [22]	13% among colorectal cancer survivors	0.211	Not all rectal cancer patients had treatment leading to stoma. Victorian weights for stoma.
**Larynx**				
Loss of speech	Eindhoven cancer registry	6%	0.20	Proportion receiving total laryngectomy.
**Melanoma of Skin**				
- Disfigurement grade I	De Vries et al [14]	25%	0.016	Lesion was on the face & Breslow thickness ≤2 mm or on the leg and arm & Breslow thickness >2 mm. GBD weight for cleft lip treated.
- Disfigurement grade II		Male: 7%, Female: 3%	0.056	Lesion was on the face & Breslow thickness >2 mm. GBD weight for other skin disease
**Breast**				
Mastectomy	European average [23]	45%	0.20	Provisional weights
**Cervix uteri**				
- Primary infertility	Hysterectomy is the standard treatment for this cancer	<40 years: 100%	0.18	Standard treatment including hysterectomy.
- Secondary infertility		40-50 years: 100%	0.10	Provisional weights
**Corpus uteri cancer**				
- Primary infertility	Hysterectomy is the standard treatment for this cancer	<40 years: 100%	0.18	Standard treatment including hysterectomy.
- Secondary infertility		40-50 years: 100%	0.10	Provisional weight
**Ovary**				
- Primary infertility	Hysterectomy is the standard treatment for this cancer	<40 years: 100%	0.18	Standard treatment including hysterectomy.
- Secondary infertility		40-50 years: 100%	0.10	Provisional weight
**Prostate**				
- Incontinence	Kvale R et al [15],	5%	0.157	
- Impotence	Johansson et al [16]	10%	0.195	
- Primary infertility		<40 years: 25%	0.18	Infertility due to prostatectomy. Impotence has a higher weight so we assumed only 15% had disability due to infertility
- Secondary infertility		40-60 years: 25%	0.10	Provisional weight
**Testis**		16%		
- Primary infertility	Brydoy M et al [17]	<40 years: 34%	0.18	
- Secondary infertility		40-60 years: 27%	0.10	Provisional weight
**Bladder** (therapy removes prostate)				
- Incontinence	Fossa S et al [18]	5%	0.157	
- Impotence	Hardt [19]	10%	0.195	
- Primary infertility		<40 years: 16%	0.18	Infertility due to prostatectomy. Impotence has a higher weight so assumed only 15% had disability due to infertility
- Secondary infertility		40-60 years: 16%	0.10	Provisional weight

Data source for weights: Dutch and Victorian burden of disease study and the Global Burden of Disease project [3,5,6].

### Duration of different phases of disease

#### *Time to death (T*_*D*_)

The median survival time of those who will die as a consequence of one of 13 cancers (oropharyngeal, oesophageal, stomach, colorectal, liver, gallbladder, pancreatic, lung, ovarian, kidney, bladder, central nervous system cancers and leukemia), as reported by Småstuen et al [13], was used. As this could not be estimated for other sites, we assumed the following times (in parentheses) for the specific cancer sites: non-Hodgkin lymphoma (5 years), cancers of the larynx, Kaposi sarcoma, cervix uteri, corpus uteri, testis, thyroid, Hodgkin lymphoma and multiple myeloma (1 year). Time to death (Table 2) was assumed to be a biological constant that was the same in all countries.

**Table 2:** Estimates of time to cure (years), time to death (years), and time for diagnosis and treatment (years) according to cancer, as applied to all countries

**Cancer sites**	**Time to cure**	**Time to death**	**Time for diagnosis and therapy**
Lip oral cavity	7.00	3.00	0.58
Nasopharynx	7.00	3.00	0.58
Other pharynx	7.00	3.00	0.58
Oesophagus	4.00	0.70	0.50
Stomach	8.00	0.60	0.50
Colorectum	7.00	1.60	1.08
Liver	5.00	0.40	0.50
Gallbladder	5.00	0.70	0.33
Pancreas	4.00	0.40	0.50
Larynx	5.00	1.00	0.50
Lung	6.00	0.60	0.50
Melanoma of the skin	5.00	1.00	0.08
Kaposi sarcoma	0.00	1.00	0.50
Breast	7.00	5.70	0.92
Cervix uteri	4.00	1.00	0.42
Corpus uteri	6.00	1.00	0.67
Ovary	8.00	2.40	0.67
Prostate	10.00	6.70	0.58
Testis	2.00	1.00	0.42
Kidney	5.00	2.70	0.42
Bladder	4.00	2.20	0.33
Brain, nervous system	5.00	1.90	0.50
Thyroid	2.00	1.00	0.42
Hodgkin Lymphoma	6.00	1.00	0.92
Non- Hodgkin Lymphoma	5.00	5.00	0.92
Multiple myeloma	5.00	1.00	1.00
Leukaemia	4.00	1.80	1.00

Data sources: (1) Time to cure and time to death: Cancer Registry of Norway [13,24], the survival of cancer patients diagnosed in Nordic countries in 1964-2003 [25-29] and (2) Time for diagnosis and therapy: Clinical guideline for cancer treatment and care [21].

#### Time to cure (T_C_)

Estimates of the time to cure were obtained by means of visual inspection of cancer-specific relative cumulative and conditional survival curves obtained from cancer survival report of the Cancer Registry of Norway in 2007 [13]. We assumed that cure is attained when the conditional relative survival curve reaches above 90% and stabilizes (towards its asymptote). The time to cure was assumed to be a biological constant, and thus fixed across all countries (Table 2).

#### Duration of diagnosis and treatment (L_D_)

A general delay of 2 months, prior to treatment onset, was assumed. This includes a period of one month for cancer diagnosis, an initial period that includes diagnostic work-up and ascertainment of stage and degree of dissemination of the disease. In many countries, delivery of cancer treatment may be delayed up to a few weeks or months after diagnosis [30] e.g. median time between diagnosis and treatment for surgical and radiotherapy of rectal cancer patients in the Netherlands is reported as between 18 and 30 days [31]; we assumed a general delay of 1 month.

Evidence-based guidelines for cancer treatment by site in the Netherlands were examined to determine the duration of each treatment modality [21]. An exception to this rule was treatment of melanoma of skin, for which we assumed a period of one month for diagnosis and treatment (including possible delays to the latter). The time period for diagnosis and treatment (*L*_*D*_) was assumed to be constant across all countries (Table 2).

#### Disability weights

Disability weights reflect the social preference or value attached to different states of health. In calculating DALYs, the average population weight (or preference) was used, instead of individual values. The weights were estimated using the person trade-off method whereby participants were asked to value the severity of various conditions on a scale of 0 (full health) to 1 (a health state equivalent to death) relative to a set of pre-determined weights of several conditions [3]. Disability weights for each phase of the natural history of cancer by cancer site were derived from Dutch and Victorian burden of disease studies, as well as earlier estimates from the global burden of disease project [3,5,6] (Tables 1 and 3).

**Table 3:** Disability weights for each disease state according to cancer site

**Cancer sites**	**Disease states**			
	**Diagnosis & initial treatment**	**Control**	**Pre-terminal**	**Terminal**
Lip oral cavity	0.56	0.37	0.90	0.93
Nasopharynx	0.56	0.37	0.90	0.93
Other pharynx	0.56	0.37	0.90	0.93
Oesophagus	0.56	0.37	0.93	0.93
Stomach	0.53	0.38	0.93	0.93
Colorectum	0.43	0.20	0.83	0.93
Liver	0.43	0.20	0.83	0.93
Gallbladder	0.43	0.20	0.83	0.93
Pancreas	0.43	0.20	0.83	0.93
Larynx	0.56	0.37	0.90	0.93
Lung	0.72	0.47	0.91	0.93
Melanoma of skin	0.26	0.19	0.81	0.93
Kaposi Sarcoma	0.51	0.14	0.83	0.93
Breast	0.54	0.26	0.79	0.93
Cervix uteri	0.43	0.20	0.75	0.93
Corpus uteri	0.43	0.20	0.75	0.93
Ovary	0.43	0.20	0.75	0.93
Prostate	0.27	0.18	0.64	0.93
Testis	0.27	0.18	0.64	0.93
Kidney	0.27	0.18	0.64	0.93
Bladder	0.27	0.18	0.64	0.93
Brain, nervous system	0.68	0.18	0.75	0.93
Thyroid	0.27	0.18	0.64	0.93
Hodgkin lymphoma	0.55	0.19	0.75	0.93
Non-Hodgkin lymphoma	0.55	0.19	0.75	0.93
Multiple myeloma	0.19	0.19	0.75	0.93
Leukaemia	0.55	0.19	0.75	0.93

Data source: Dutch and Victorian burden of disease study and the Global Burden of Disease project [3,5,6].

Estimates applied to all countries.

#### Discounting and age weighting

Controversies exist as to whether discounting should be applied when DALYs are measured [32]. Earlier studies of the burden of disease have applied a discount to the future years lost, so to give more weight to lost years that occur nearer to the present time [3]. To study the effect of time discounting on the estimated of DALYs, we applied a 3% time discount rate [7]. To give more relevance to deaths in young and middle age, we also tested the use of non-uniform age weights, we used the standard formula:

(4) $$X_{w}={C_{x}}^{-\beta x}$$

where *X*_*w*_ is the weighted age (years), *C* and *β* are constant and *x* is age (in years) [3].

### Sensitivity analyses

We examined the impact on the estimate of DALYs of several different assumptions in the calculation:

1. Estimates of the proportion cured and proportion treated by country. Estimates from Norway, based on the reported proportion cured and proportion treated from the Cancer Registry of Norway, were compared with those derived through the modeling exercises, described above. We also validated the modeled estimates of proportion treated against observed treatment data obtained from eight Cancer Registries (Bulgaria, India (Mumbai), Ireland, Jordan, Norway, Poland (Holy Cross), the Republic of Korea and Slovakia).

2. Use of a two-stage natural history model. We sought to assess the impact on the DALYs if a simpler two-stage model, in which data on treatment is no longer required, was used (Figure 1b). In this model, all patients are assumed to go through the diagnosis and treatment phase. As most cancer registries do not have complete and accurate data on the nature of cancer treatment (curative or palliative), or cancer-specific treatment (surgery, radiotherapy etc), the two-stage disease model has its advantages. Patients who eventually are cured (*s*) went into a remission phase before cure or continue to live with sequelae. The second (uncured) group went first into remission, then pre-terminal and terminal phases and eventually died from cancer.

3. Using the reported proportion of advanced cancer cases as a proxy for the proportion that did not receive treatment. The lack of high-quality treatment data worldwide is a concern in calculating DALYs and we assessed whether the proportion of cases with distant cancer is a better proxy for the proportion of cases who did not receive curatively-intended treatment in the three-stage natural history disease.

4. Impact of discounting and age weighting (as described above).

In the text that follows we report the results of each of these validation exercises the estimated DALYs in four countries (Norway, Bulgaria, India and Uganda), populations representative of the four quartiles of the human development index (very high, high, medium and low, respectively). The DALYs are presented in these countries, examining the relative contribution of YLL and YLD to the DALYs according to cancer site.

## Results

### Estimates in four countries: Disability-adjusted live years

**Figure 2:**
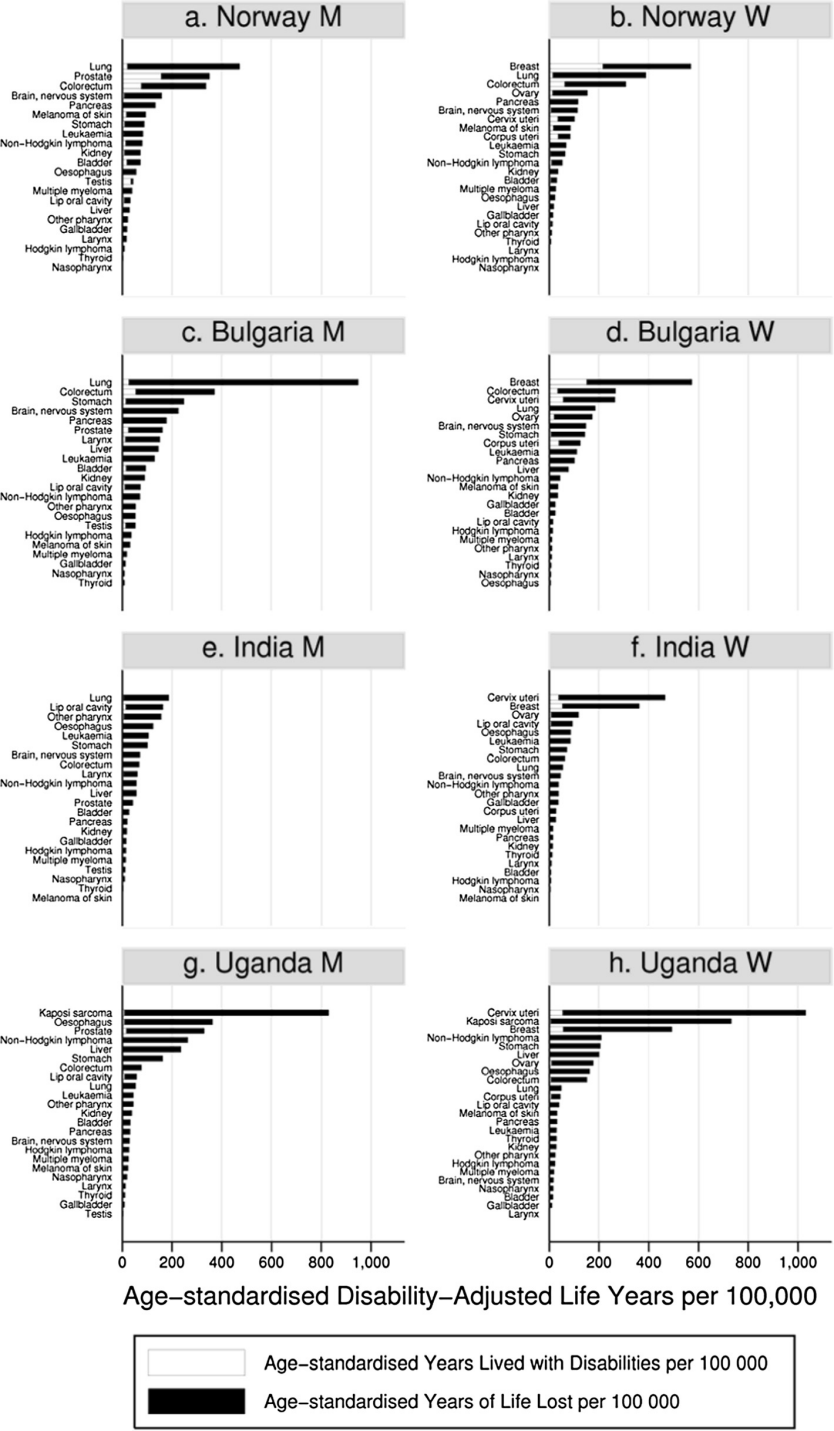
**Years lost due to disability (YLDs, white bar), years of life lost (YLLs, black bar) and disability-adjusted life years (DALYs, sum of YLLs and YLDs) per 100,0000 in 2008 for Norway (a and b), Bulgaria (c and d), India (e and f) and Uganda (g and h).** Estimates were age standardised according to the world standard population and not discounted nor weighted (0,0).

In the four countries considered (Norway, Bulgaria, India and Uganda) the total DALYs lost due to the 27 cancer sites included in this study were 4503, 5569, 3022, and 6491 per 100,000 population respectively (Figure 2). The main differences between countries were: (1) Rankings of the various cancer types and (2) Proportion of YLDs or YLLs over DALYs. Cancer of the lung, colorectum, prostate, breast and cervix uteri contributed to the largest lost in DALYs in Norway or Bulgaria. In India besides lung, breast and cervical cancers, cancer of the oral cavity was the main cause of lost in healthy years. In Uganda, HIV/AIDS defining cancers such as Kaposi sarcoma, non-Hodgkin lymphoma and cervical cancers were identified as making the largest contribution to DALYs, in addition to oesophageal and prostate cancer for men and breast cancer for women.

In all countries most of the DALYs lost are due to early death from cancers i.e. YLLs over DALYs were 81%, 90%, 94% and 97% in Norway, Bulgaria, India and Uganda. The YLDs had more importance in cancers that are both common (high incidence) and with moderate or good survival, such as breast, colorectum, prostate and cervical cancers. Generally, the fraction of DALYs represented by YLDs was greater in the more developed countries. For example for breast cancer, the proportions of YLDs over DALYs were 38%, 26%, 15% and 11% in Norway, Bulgaria, India and Uganda respectively. The proportions of YLDs over DALYs were also substantial for testicular cancer (79% in Norway) and Hodgkin lymphoma (55% in Norway).

### Sensitivity analyses

#### Observed versus modeled estimates of the proportion cured/proportion treated and impact on DALYs

**Figure 3:**
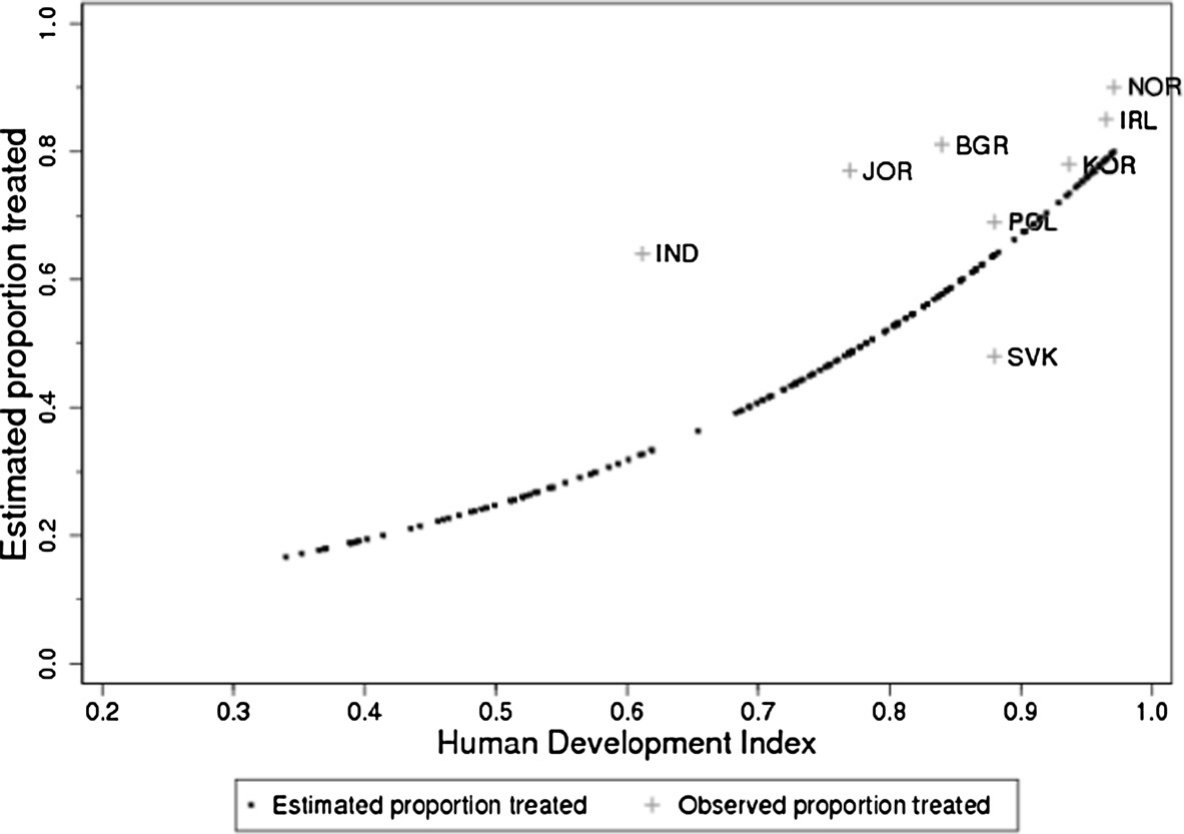
Observed proportion treated in 8 cancer registries and estimated proportion worldwide for colorectal cancer.

We validated the model-based estimates of proportion cured and treated by comparing these with the observed data from Norway (Table 4). Additionally, the model-based country-specific estimates of the proportion treated were also validated by comparing these with observed data from eight registries. The proportion cured and treated estimated using our proposed method gave a reasonably good fit with the observed values in Norway. As for the other nations, the model-based estimates for proportion treated were in line with those observed for good prognosis cancers including colorectum (Figure 3), larynx, breast, cervix, prostate, testis, kidney, bladder, thyroid and melanoma of the skin. While the model fit was also acceptable for some poorer prognosis cancers including lung, ovary, gallbladder and brain and nervous system. Because an inverse in the relation between human development index and proportion treated was observed for pancreatic and liver cancer, we decided to take the treatment proportion observed in Norway for all countries. This is probably caused by the very poor survival of patients with these cancers causing an M:I ratio ~ 1.

**Table 4:** Observed (O) and estimated (E) values of proportion cured and treated, and age standardized disability-adjusted life years (DALYs) per 100,000 population, by cancer in Norway in 2008 derived from observed estimates of the proportion cured and proportion treated, versus expected values derived from models

**Cancer sites**	**Proportion cured (%)**		**Proportion treated (%)**		**DALYs**^a^	
	**O**	**E**	**O**	**E**	**O**	**E**
Lip oral cavity	39	33	91	77	46	45
Nasopharynx	39	35	91	81	3	3
Other pharynx	39	39	91	91	32	32
Oesophagus	11	10	52	46	80	79
Stomach	21	34	46	72	149	153
Colorectum	56	50	90	81	658	644
Liver	10	10	30^b^	30^b^	48	48
Gallbladder	16	38	34	79	34	34
Pancreas	6	5	27^b^	27^b^	248	248
Larynx	66	48	87	63	21	20
Lung	12	10	17	14	864	860
Melanoma of skin	80	76	95	90	181	180
Kaposi Sarcoma ^c^	0	0	0	0	0	0
Breast	76	63	98	81	609	569
Cervix uteri	74	59	87	70	109	101
Corpus uteri	80	71	95	85	88	84
Ovary	35	30	83	72	155	153
Prostate	69	64	79	73	362	351
Testis	96	96	99	99	43	43
Kidney	47	46	80	79	108	108
Bladder	67	62	94	87	105	104
Brain, nervous system	64	26	83	34	282	272
Thyroid	89	89	95	95	11	11
Hodgkin lymphoma	86	90	91	95	11	11
Non-Hodgkin lymphoma	40	37	74	69	134	132
Multiple myeloma	37	34	68	62	66	66
Leukaemia	51	35	47	32	153	152

^a^Not discounted with equal age weight.

^b^Observed values were used to estimate DALY because inadequate fit of estimated values.

^c^Incidence and mortality in Norway from Kaposi Sarcoma was 0.

Finally Table 4 shows age-adjusted DALY rates for Norway using the model-based estimates of the proportion cured and the proportion treated, compared with those calculated using observed values for these variables. The impact of using estimated values on the overall DALYs was negligible, particularly for cancers of the oesophagus, colorectum, endometrium, cervix, prostate, testis, kidney, bladder, thyroid and Hodgkin lymphoma. More sizable differences were observed for cancers of the breast, cervix uteri, corpus uteri and larynx (ranging from 5-9% difference to the main results where estimates were modeled).

#### Two- versus three-stage natural history models

The age adjusted DALYs per 100,000 for Norway, estimated by the two-stage history model, are presented in Table 5 (sensitivity analysis 1), and compared with the observations using the 3-stage model (see Table 4). Using this model and assuming all cancers patients were treated, hardly affected the total DALY rates for most cancer sites. Estimates for the DALYs were generally larger than those derived from the three-stage disease model for good prognosis cancers such as breast and prostate cancer. The percentage difference in DALY rates, as compared to the three-stage disease model ranged from 0% to 9%.

**Table 5:** Sensitivity analyses, age standardised disability-adjusted life years (DALYs) per 100,000 population, and percentage difference (%Diff) as compared to the main analysis, Norway 2008

**Cancer sites**	**Sensitivity analysis 1**^**a**^		**Sensitivity analysis 2**^**b**^	
	**DALYs**	**%Diff**	**DALYs**	**%Diff**
Lip oral cavity	46	2	46	2
Nasopharynx	3	0	3	0
Other pharynx	33	3	33	3
Oesophagus	80	1	80	1
Stomach	152	0	152	−1
Colorectum	643	1	643	0
Liver	48	0	48	0
Gallbladder	34	0	34	0
Pancreas	248	0	248	0
Larynx	20	5	- ^c^	- ^c^
Lung	864	1	864	0
Melanoma of skin	180	1	180	0
Kaposi Sarcoma	-^d^	-^d^	-^d^	-^d^
Breast	587	4	587	3
Cervix uteri	102	1	102	1
Corpus uteri	84	1	84	0
Ovary	151	1	- ^e^	- ^e^
Prostate	364	9	364	4
Testis	44	0	- ^e^	- ^e^
Kidney	110	2	108	0
Bladder	105	1	105	1
Brain, nervous system	281	3	- ^c^	- ^c^
Thyroid	11	0	- ^e^	- ^e^
Hematological cancers	- ^f^	- ^f^	- ^f^	- ^f^

^a^ Sensitivity analysis 1: Two-stage disease model as compared to three stage disease model.

^b^ Sensitivity analysis 2: Proportion of advanced cases used as proxy of patients who did not receive (curatively intentioned) treatment as compared to use of available treatment data.

^c^ Staging data were not available.

^d^ Treatment and staging data were not available.

^e^ Proportion cured was larger than proportion of advanced cases, analysis was not done.

^f^ Sensitivity analyses 1 and 2 were not done for haematological cancers.

#### Advanced cancers as a proxy for untreated proportions

Table 5, sensitivity analysis 2, shows the effect on the estimate of DALYs in Norway of using the observed proportion of advanced cancers as a proxy for the proportion treated. The largest difference in DALYs was only observed for breast and prostate cancers, increasing the estimates by 4% respectively, suggesting an overestimation of DALYs, probably the result of overestimating the proportion of patients who received curative treatment. For thyroid, testicular and ovarian cancers, the proportion of advanced cases was larger than the proportion uncured so we did not perform this analysis for these cancers. The stage distribution was not reported or was not available for laryngeal, brain and nervous system as well as haematological cancer, and was therefore omitted from the analysis.

#### Impact of discounting and age weighting

As expected, compared with the basic model the rates of DALYs after discounting and age weighing are much lower. Discounting and age weighing did not substantially change ranking of cancer sites. Generally we observed an increase in the proportion of DALYs due to YLDs after discounting and age weighting. The size and direction of the effect varied by country, but the increase in the YLDs:DALYs ratio was greatest in Uganda, and generally larger for cancers of the brain, nervous system and Hodgkin Lymphoma.

## Discussion

In this paper we propose a methodology for estimation of global disability-adjusted life years for cancer. Many epidemiological variables are required to compute DALYs in a single country according to the three-stage natural history model, and, given the paucity of such data (irrespective of quality) in many countries, the compilation of DALYs at the global level is particularly challenging. Variables such as the proportion cured from, or treated for different cancers, required in the calculation of the YLDs, are unavailable in most countries, particularly in low and middle income regions. The approach outlined in this paper has produced a practical set of estimates enabling cross-country comparisons of DALYs and their two components, YLL and YLD. Such indicators – over and above incidence, mortality and survival - provide valuable additional information for planning and investing in cancer services within current health systems and help establish the need for population interventions aimed at reducing the burden of the disease.

Cancer survival has been increasing over the last four decades in many more developed countries such as Norway and Bulgaria. Inequalities in survival are reflected in the larger proportion of the DALYs that are attributable to YLD in these countries, compared to the less developed countries such as India or Uganda. Advances in the treatment of several cancers - such oral cavity, leukemia, testicular cancer has resulted in increasing survival [34]. Earlier detection of breast, cervical and colorectal cancer has increased the rate of treatable cases and thus survival in developed countries [33]. Yet facilities for cancer prevention, diagnosis and treatment in developing countries remain inadequate, calling for action to scale up these activities. In addition we also observed large rates of DALYs attributed to highly preventable cancers such as lung, oral cavity or stomach in all countries. This points to the importance of intervention to reduce cigarette smoking. In Uganda, cancers related to infection (Kaposi sarcoma, cervix cancer, non Hodgkin lymphoma, liver cancer) make a large contribution in DALYs highlighting the value of vaccination or treatment of the various infectious diseases.

**Figure 4:**
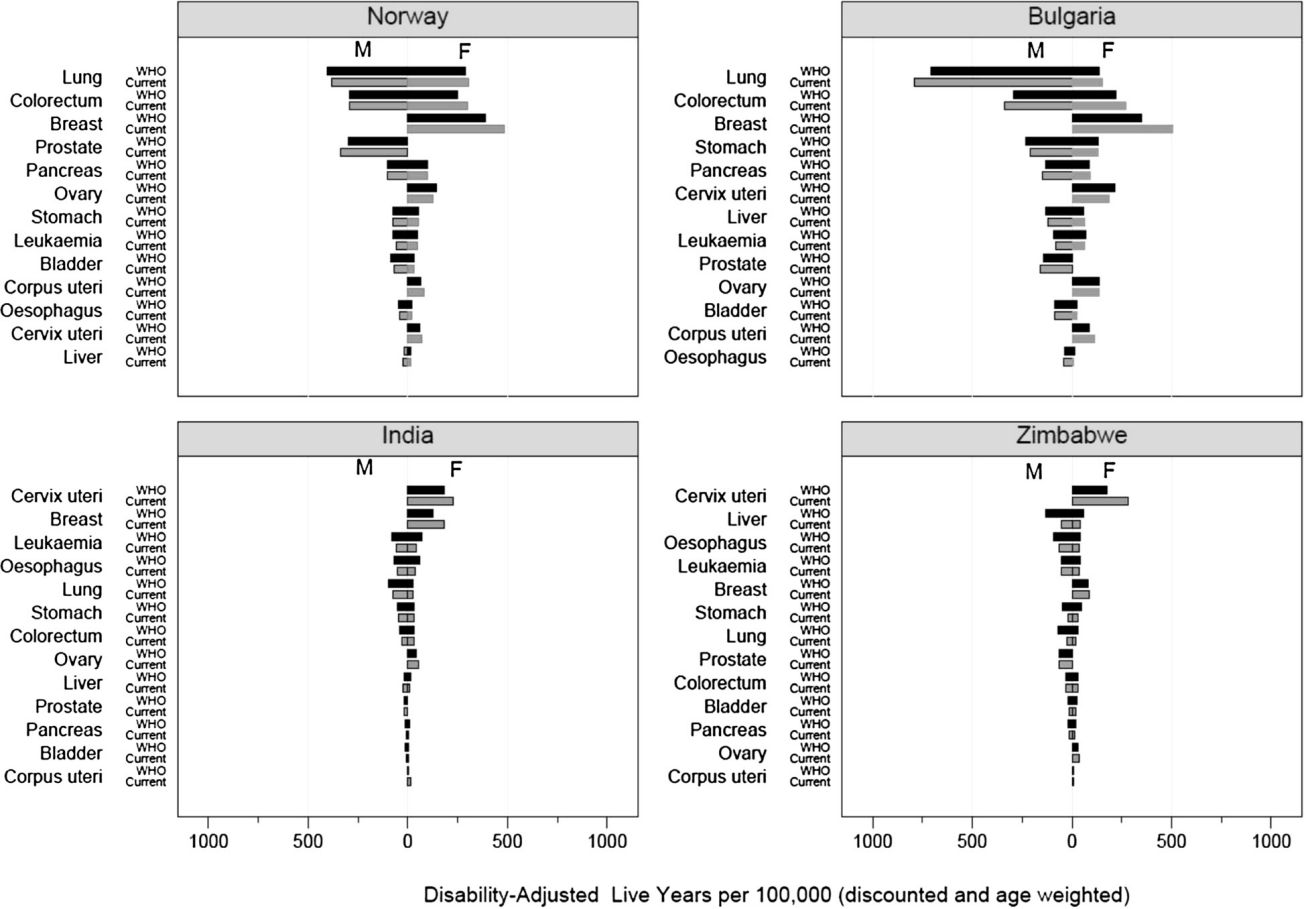
**Comparison of disability-adjusted live years per 100,000 (3% discounting and age weighting) for 13 cancers in Norway, Bulgaria, India and Uganda, as estimated in the present study (current), and in the GBD study of WHO (WHO) **[1].

Previous studies of the global burden of cancer were performed by WHO as part of a wider effort to map the global burden of disease in general [1,3]. This project, commissioned in 1990 [3], introduced DALYs as a means of facilitating comparison across diseases, countries and over time. In 2004, WHO published disease- and country-specific DALYs [1], and their estimates are generally comparable to our current estimates (as illustrated in Figure 4). Some differences can be attributed to changing rates of incidence or mortality for some cancer sites, as observed for lung cancer in Norway (decreasing) and in Bulgaria (increasing) [35]. The DALY rates for breast, cervical and prostate cancer were relatively high in the present study, most likely due to the improvement in the YLD calculation, by incorporating a more detailed disease quantification of phases and sequelae.

This project has been followed by more recent assessments of the global disease burden, as well as various national initiatives [36-40]. These studies are mostly confined to more developed countries, where similar observations to our findings have been noted, with lung, colorectal, and breast cancers sharing the largest proportion of DALYs. Our study was able to draw on more current epidemiological data to derive incidence and mortality estimates [2,41], population-based data on cancer specific-treatment and outcomes including survival, on the basis of more recent reviews [15-17,19]. A major improvement in this study relative to previous studies is the use of observed survival data in estimating the proportion cured and the median survival of uncured patients [13] as a means to calibrate country-specific estimates of the proportion cured and proportion treated. In addition, larger lists of incorporated sequelae and more information on the relative proportions of treated and non-treated patients, using population based registry data, will have resulted in a more valid set of estimates than has been possible in previous exercises.

We modeled proportion cured based on the relation between the mortality to incidence ratio and the human development index. In an earlier paper we have shown a strong correlation between gross domestic product (GDP) and cancer specific survival [2]. In this paper, HDI was chosen over GDP because HDI that also covers wealth, health and education [12] gave better fit than GDP to survival in our internal analysis. This was confirmed on establishing reasonably well fitting models and predicted estimates for the proportion cured on the basis of HDI. The modeled proportion treated also corresponded well to that reported by several cancer registries. Finally the estimated DALYs in Norway based on the modeling approach were very similar to those calculated using observed data from the Registry, serving as further indication of the validity of proportion cured and proportion treated based on the former procedures.

The simpler two-stage [36] natural history model was compared with the three-stage model to calculate DALYs, in view of the principle that it may reduce the complexity of the data required, and calculations. When the two-stage model was used, it generally increased the DALYs, although the differences with the three-stage disease model were rather modest. In the two-stage disease model all patients received curative-intended treatment, and thus all patients who would eventually die from cancers went through remission followed by pre-terminal and terminal phases. In this model, the increase in time to death increased YLD. Therefore for cancers with long remission times (for example, prostate cancer), the differences between the two- and three-stage models becomes larger.

As the intention of treatment (curative or palliative) tends to be recorded less well than treatment modality, we assumed that the proportion of patients receiving any cancer treatment was a reasonable proxy of proportion curatively treated. This is probably a slight overestimation of the true proportion of patients who received curative treatment, as was indicated by the sensitivity analysis. We also considered the use of advanced cases as a proxy of patients who did not receive curative treatment; this had an effect of overestimating the YLD by up to 4%. Such an observation likely resulted from a larger proportion of YLD contributed by patients who were treated but eventually died from cancer.

The limitations of this study pertain to the inputs and necessary assumptions we have made, given the lack of information available at the appropriate level of detail as input for the calculation of DALYs. Firstly incidence and mortality data were derived from the GLOBOCAN2008 which are estimates with varying accuracy depending on the availability of country-specific data [2]. In most low-income countries, there are no comprehensive national-level data on cancer incidence and mortality, and the estimates are based on model-based rates of mortality (from WHO) and/or limited data on cancer incidence from regional cancer registries.

Secondly, the YLD calculations are based on rather limited country-level data, and most parameters are based on data from high-income countries. As an example country-specific proportion cured was modeled using survival:proportion cured ratio as observed in Norway. Applying this ratio to other higher income country may not cause substantial bias as treatment and follow-up practices may be comparable. Third line treatments might delay deaths from relatively advanced disease and hence after “statistical” cure, patients may still have a higher mortality relative to that observed in the general population. On the other hand, it may be hypothesised in less developed countries, treated patients who are considered cured effectively have a similar mortality experience to the general population. In such circumstances, we may underestimate the proportion cured in the lower and middle income countries.

Furthermore, we assumed a uniform period of two months between onset of symptoms and treatment (allowing for delays due to the patient and the medical care system). While the duration of delay might be reasonable for high income countries, it is probably too optimistic in low and middle income settings. While there are many population-specific studies related to specific cancers [42-44], we are not aware of any overall comparative assessment. In any case, a longer period of disability in this phase is likely to be offset by shorter pre-terminal and terminal phases: and the contributions of YLDs from these disease phases comprise a very small proportion of the cancer-specific DALYs in lower income countries.

Fourthly, in calculating DALYs, we clearly missed many long-term consequences from cancers. For example infertility after chemotherapy for haematological cancer patients is well reported [45], but we assumed that these patients were completely cured from cancer. In addition to the observable clinical sequelae many survivors continue to live with psychological stress related to their cancer diagnosis [46]. The proportion living with sequelae is calculated based on studies and data from developed countries that may approximate the proportion in countries with similar level of development. Yet, in addition to low availability and access to modern cancer care, in developing countries cancer cases show a less favorable stage distribution that requires more aggressive treatment, hence a higher proportion of survivors with cancer sequelae. Suboptimal treatment and follow-up for cancer patients in less developed countries may also cause higher levels of disability, and yet we assumed similar disability weights for each sequelae in all countries. Taking into account of these factors are important in future studies where the burden and hence the priorities in cancer control can be better determined.

Finally, the burden of disease study in Australia has adjusted disability weight for common co-occurring diseases, which we have not undertaken in this study [5]. Because we have assessed morbidity and disability due to cancer only, the disability due to co-existing disease is not considered in the analysis. In previous study where burden from multiple diseases were estimated [5], a down-weighting of the comorbidity avoided multiple-counting of morbidities. For cancer the vast majority of comorbidities will be less severe (e.g. arthritis, vision problems etc.) and the comorbidity adjustments will not be very large. For severe comorbidities where the adjustment may be large, these will be very rare.

## Conclusions

In this paper we have presented a methodological approach to derive the variables needed to compute DALYs from cancer on a global scale. Such estimates should help setting global priorities in health care with a view to reducing the burden of cancer. The methodological approach suggested in this study should enable all countries worldwide to calculate DALYs from cancer with a reasonable degree of validity. Greater precision in the sets of parameters used in this study would be provided for by an enhancement of data availability and accuracy, and the reporting of the relevant statistics required.

Based on the results of this study, it highlights the important role of prevention in reducing the cancer burden given the large share of premature mortality from cancer, as estimated in the DALYs; even in a country with high-quality cancer services such as Norway. Taking a long-term perspective, a continuous global effort to increase the awareness of prevention and treatment is needed. This includes the provision of estimate of DALYs as a means to assess the need for improvements in early detection and treatment, thus reducing the existing inequalities in the availability and access to cancer diagnostics and care.

## Competing interests

The authors declare that we have no competing interests.

## Authors' contributions

IS contributed to the data collection, study design, analysis and wrote the first draft of the paper. JT contributed to the study design, analysis and finalising the manuscript. JF contributed to the data collection and finalising the manuscript. DF contributed to finalising the manuscript. DMP and CM contributed to the study design and finalising the manuscript. FB contributed to the study design and analysis and in drafting the manuscript. All authors read and approved the final manuscript.

## Pre-publication history

The pre-publication history for this paper can be accessed here:

http://www.biomedcentral.com/1471-2288/12/125/prepub
